# Solid organ transplantation and gut microbiota: a review of the potential immunomodulatory properties of short-chain fatty acids in graft maintenance

**DOI:** 10.3389/fcimb.2024.1342354

**Published:** 2024-02-27

**Authors:** Manon Jardou, Clarisse Brossier, Pierre Marquet, Nicolas Picard, Anne Druilhe, Roland Lawson

**Affiliations:** National Institute of Health and Medical Research (FRANCE) (INSERM), Univ. Limoges, Pharmacology & Transplantation, U1248, Limoges, France

**Keywords:** solid organ transplantation, graft rejection, immunosuppressive drugs, gut microbiome, short-chain fatty acids, immune system modulation

## Abstract

Transplantation is the treatment of choice for several end-stage organ defects: it considerably improves patient survival and quality of life. However, post-transplant recipients may experience episodes of rejection that can favor or ultimately lead to graft loss. Graft maintenance requires a complex and life-long immunosuppressive treatment. Different immunosuppressive drugs (*i.e.*, calcineurin inhibitors, glucocorticoids, biological immunosuppressive agents, mammalian target of rapamycin inhibitors, and antiproliferative or antimetabolic agents) are used in combination to mitigate the immune response against the allograft. Unfortunately, the use of these antirejection agents may lead to opportunistic infections, metabolic (*e.g.*, post-transplant diabetes mellitus) or cardiovascular (*e.g.*, arterial hypertension) disorders, cancer (*e.g.*, non-Hodgkin lymphoma) and other adverse effects. Lately, immunosuppressive drugs have also been associated with gut microbiome alterations, known as dysbiosis, and were shown to affect gut microbiota-derived short-chain fatty acids (SCFA) production. SCFA play a key immunomodulatory role in physiological conditions, and their impairment in transplant patients could partly counterbalance the effect of immunosuppressive drugs leading to the activation of deleterious pathways and graft rejection. In this review, we will first present an overview of the mechanisms of graft rejection that are prevented by the immunosuppressive protocol. Next, we will explain the dynamic changes of the gut microbiota during transplantation, focusing on SCFA. Finally, we will describe the known functions of SCFA in regulating immune-inflammatory reactions and discuss the impact of SCFA impairment in immunosuppressive drug treated patients.

## 1 Introduction

Transplantation is the treatment of choice for end-stage failure of several organs. In 2020, according to the Global Observatory on Donation and Transplantation, kidney was the most commonly transplanted organ worldwide followed by liver (World Health Organization, 2022). In 2022, kidney and liver accounted for, respectively, over 60% and 25% of transplanted organs. Organ transplantation improves not only patient survival but also their quality of life (Grinyó, 2013; Black et al., 2018). Over the past 40 years, the global graft survival rate following solid organ transplantation has improved considerably, due to advances in immunosuppressive therapy. In France in 2021, graft survival was 91% at one year, but only 60% at ten years post-transplantation (Agence de la biomédecine, 2021). Indeed, even though they are receiving immunosuppressive drugs, transplant recipients may experience episodes of graft rejection that can precipitate graft loss. In addition, immunosuppressive drugs have adverse effects that limit the improvement in quality of life of some patients (Ruiz and Kirk, 2015). In road to precision medicine in transplantation, it is important to control the factors that contribute to variability in the therapeutic response to immunosuppressants. One of the factors of variability is the gut microbiota, whose fundamental role in regulating the immune system has been recently highlighted (Zhang et al., 2019).

Actually, there is no direct evidence for changes in gut microbiota metabolites and graft rejection or even the development of comorbidities in transplant patients. Although there is still a need for rigorous studies to fill this knowledge gap, converging data and a weight of evidence suggest that short-chain fatty acids (SCFA) derived from the gut microbiota may contribute to the common comorbidities (cardiovascular and metabolic disorders) in transplant patients and to graft rejection. For example, an alteration in SCFA-producing bacteria, mainly related to the immunosuppressive protocol, has been reported in kidney transplant patients (Swarte et al., 2020). In addition, an alteration of SCFA profile in mice treated with mycophenolic acid, the most commonly used immunosuppressive drug, has been observed (Jardou et al., 2021). SCFA influence the host homeostasis and are involved in gut barrier integrity (Liu et al., 2021), regulation of glucose and lipid metabolism (Chambers et al., 2018), and control of inflammatory responses and activation of the immune system (Zhang et al., 2019).

Herein, after a brief overview of the mechanisms of graft rejection prevented by immunosuppressive drugs, we will thoroughly discuss the changes that the gut microbiota may undergo because of immunosuppression during transplantation, with a focus on gut microbiome-derived short-chain fatty acids (SCFA). Finally, we will present the physiological role of SCFA in modulating the immune system and we will discuss the consequences of SCFA changes in immunosuppressant-treated transplanted patients.

## 2 Graft rejection mechanisms

**Figure 1:**
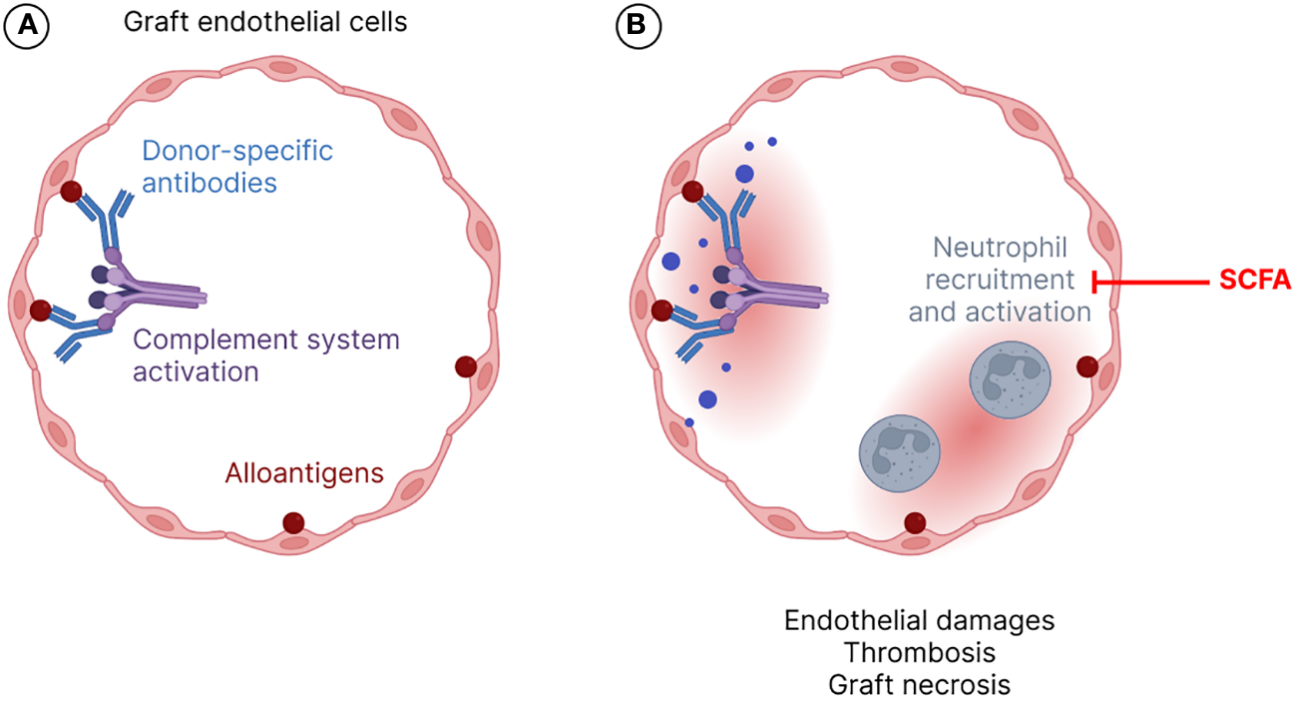
Hyperacute rejection mechanism. Alloantigens, *i.e.*, donor-specific antibodies (DSAs), bound to endothelial cells of the grafted organ vessels, induce complement activation **(A)** and neutrophil recruitment and activation **(B)**. All these processes contribute to endothelial damage, thrombosis, and graft necrosis. SCFA stands for short chain fatty acids.

The innate and adaptive immune systems are involved in allograft rejection (Naik and Shawar, 2020). Several types of rejection, i.e. hyperacute, antibody- or T cell-mediated, acute or chronic processes have been described, depending on histopathology and immunological characteristics as well as on the time course of rejection (Moreau et al., 2013). Hyperacute rejection occurs in the first minutes to hours after transplantation and is due to a preformed antibodies that react with alloantigens (*e.g.*, ABO blood type antigens, major histocompatibility complex -MHC- antigens) present on the surface of grafted cells leading to endothelial damage, platelet accumulation and thrombosis in capillaries (Solez et al., 1993). Indeed, pre-existing donor-specific antibodies (DSA) in the recipient induce complement system activation and massive inflammatory response with recruitment and activation of polymorphonuclear neutrophils leading to thrombosis, ischemia and graft necrosis (Lala et al., 2014) (**Figure 1**). Nowadays, this type of graft rejection is very rare due to prior investigations of tissue compatibility between the donor and the recipient (Tittelbach-Helmrich et al., 2014). During the first weeks or months after transplantation, the graft can undergo acute rejection episodes by two distinct mechanisms: the B-cell dependent pathway that results in antibody mediated rejection (ABMR), also known as humoral response or antibody-mediated rejection, which refers to microvascular inflammation following B-cell activation, plasma cell differentiation and production of antibodies targeting the donor endothelium; and/or the T-cell dependent pathway that corresponds to T-cell mediated rejection (TCMR), resulting from tubulointerstitial inflammation following T-cell activation and migration into the allograft (Moreau et al., 2013; Loupy et al., 2020). For kidney transplants, ABMR and TCMR are diagnosed based on elementary histological lesions on graft biopsies, interpreted according to the Banff international classification system, first published in 1993 and regularly revised (Solez et al., 1993; Loupy et al., 2020).

**Figure 2:**
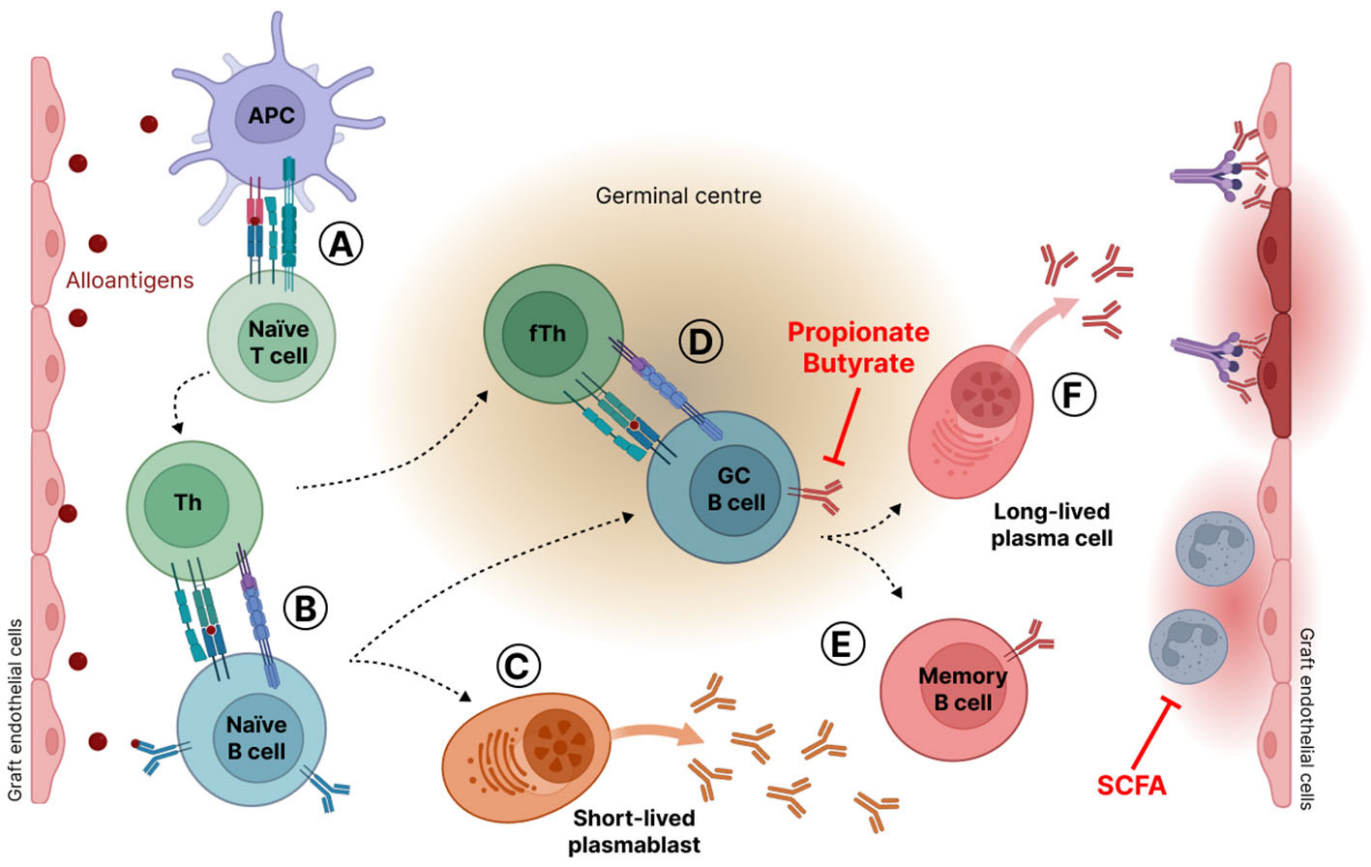
Antibody mediated rejection mechanism (ABMR). **(A)** Alloantigens from damaged allograft tissue activate antigen presenting cells (APC). Activated APC present antigens bound to the major histocompatibility complex (MHC) to naïve T lymphocytes that differentiate into helper T (Th) lymphocytes. **(B)** Activated Th cells in turn deliver activating signals to naïve B lymphocytes. Activated B cells either differentiate in short-lived plasmablasts that produce low-affinity antibodies **(C)** or migrate into the germinal center **(D)** where they are submitted to proliferation, gene mutations, and selection through interaction with T follicular helper cells (Tfh) and follicular dendritic cells (FDC). Gene mutations lead to antibody affinity maturation and immunoglobulin class switching. Germinal center B cells are eliminated by apoptosis or maturate in memory B cells **(E)** or in long-lived plasma cells that secrete high-affinity antibodies against graft endothelial cells **(F)**. SCFA stands for short-chain fatty acids.

For the diagnosis of ABMR, different criteria must be met including histological evidence of current or recent antibody interaction with the vascular endothelium (Loupy et al., 2020). During ABMR, alloantigens from damaged allograft tissue activate APC, which in turn present antigens, through binding to MHC, to naïve T lymphocytes (Lee et al., 2020). As a result, naïve T lymphocytes differentiate into helper T (Th) lymphocytes that provide activating signals to naïve B lymphocytes. Activated B cells either differentiate in short-lived plasmablasts that produce low-affinity DSA or migrate into the germinal center (GC). In GC, activated B cells proliferate, undergo immunoglobulin gene mutation, including somatic hypermutation and immunoglobulin class switching, and are selected under the control of follicular helper T cells (Tfh) and follicular dendritic cells (FDC) (Klein and Dalla-Favera, 2008). The GC reaction leads to the death of B cells or to their maturation in memory B cells or in the generation of long-lived plasma cells that secrete high-affinity DSA (González-Molina et al., 2016). DSA target graft cells, resulting in complement activation and inflammatory reactions with the recruitment and activation of neutrophils. These phenomena induce graft tissue damage (Suchanek and Clatworthy, 2020; Oellerich et al., 2021) (**Figure 2**).

**Figure 3:**
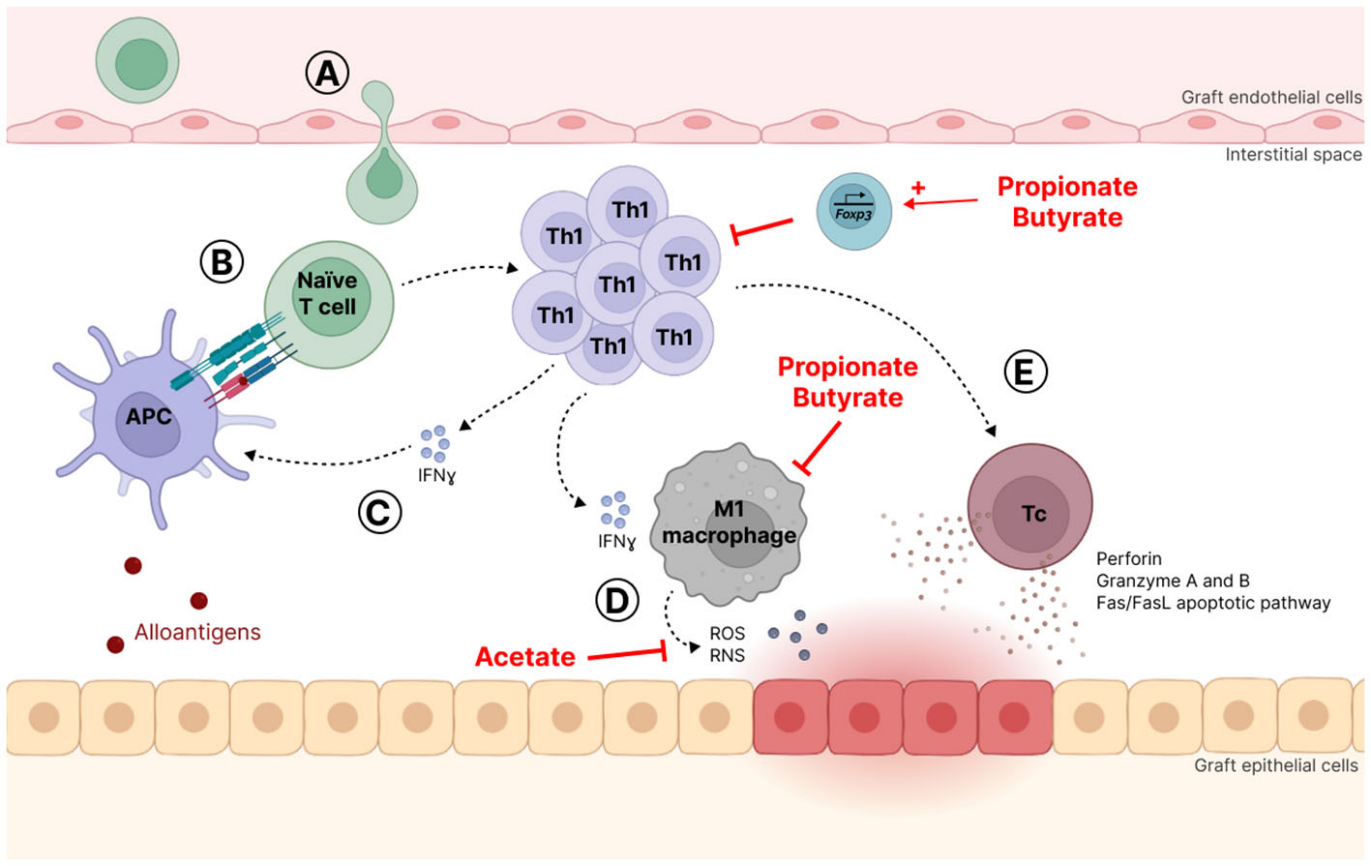
T-cell mediated rejection mechanism (TCMR). **(A)** Naïve T lymphocytes infiltrate the interstitial space where they are activated **(B)** by antigen-presenting cells (APC), which present alloantigens through binding to major histocompatibility complex (MHC). **(C)** Activated T lymphocytes proliferate and differentiate into T helper 1 (Th1) cells that produce interferon-g (IFNγ), a pro-inflammatory cytokine that activates APC. **(D)** IFNγ also promotes macrophage recruitment and polarization towards a proinflammatory phenotype called M1 that abundantly secretes proinflammatory molecules and cytotoxic products such as reactive oxygen species (ROS) and reactive nitrogen species (RNS). **(E)** Th1 also favor cytotoxic T (Tc) cell differentiation that may cause graft cell death by releasing cytolytic granules and activating the Fas/FasL apoptotic pathway.

TCMR is characterized by the accumulation of mononuclear cells, mostly Th cells, cytotoxic T lymphocytes (Tc) and macrophages, in the interstitial space of graft tissue leading to interstitial inflammation combined with inflammation of the tubules and the arteries (Loupy et al., 2020). It is believed that naïve T cells infiltrate the interstitial space and are activated by APC, either dendritic cells (DC) or macrophages, which present alloantigens through MHC binding to T cells. Afterwards, these T cells proliferate and differentiate in type 1 Th (Th1) that produce interferon-γ (IFNγ), a pro-inflammatory cytokine that in turn activates APC (Eikmans et al., 2019). IFNγ also promotes macrophage recruitment and their polarization in a proinflammatory phenotype called M1. M1 macrophages secrete abundant amounts of proinflammatory cytokines, *i.e.*, interleukin (IL)-1, IL-12, IL-6, tumor necrosis factor α (TNFα), and IFNγ (Wyburn et al., 2005; Wang et al., 2020a). Activated macrophages release also cytotoxic products such as reactive oxygen species (ROS) and reactive nitrogen species (RNS) (Panzer, 2022). Cytotoxicity against graft cells, either epithelial or endothelial, results also from the Th1-mediated differentiation of Tc. Indeed, Tc may cause death by releasing cytolytic granules containing perforin, granzyme A and B and by inducing the Fas/FasL apoptotic pathway in graft cells (Robertson et al., 1996; Cornell et al., 2008) (**Figure 3**).

Unlike acute rejection, which occurs rapidly after transplantation, chronic rejection develops slowly and progressively over a period of several months or years and is the major cause of long-term graft loss (Moreau et al., 2013). According to the Banff classification, characteristic lesions of chronic ABMR are constituted by evidence of chronic injury, *e.g.*, transplant glomerulopathy and arterial intima fibrosis, antibody action such as the presence of C4d, with or without circulating DSA (Colvin, 2007; Cornell et al., 2008; Loupy et al., 2020). The underlying mechanisms are complex and involve both adaptive and innate immune responses, failure to maintain sufficient immunosuppression and other risk factors (*e.g.*, age, overweight, hypertension) (Joosten et al., 2005). Chronic TCMR is less common and according to the Banff classification, the criteria include interstitial inflammation and fibrosis, tubulitis and tubular atrophy (Loupy et al., 2020; Mizera et al., 2023).

Lately, natural killer (NK) cells emerged as another essential actor of graft rejection. NK are activated either by alloreactive T cells or by the presence of DSA or the absence of self-markers on the surface of grafted cells. Once activated, they can orchestrate the response of other immune cells by producing cytokines including IFN-γ, and they are directly involved in graft cell damage *via* the release of cytotoxic products. Because of their properties, NK cells have been shown to contribute to acute as well as to chronic rejection processes (Pontrelli et al., 2020; Hamada et al., 2021; Miyairi et al., 2021).

## 3 Immunosuppressive drug

### 3.1 Mechanisms of action

**Figure 4:**
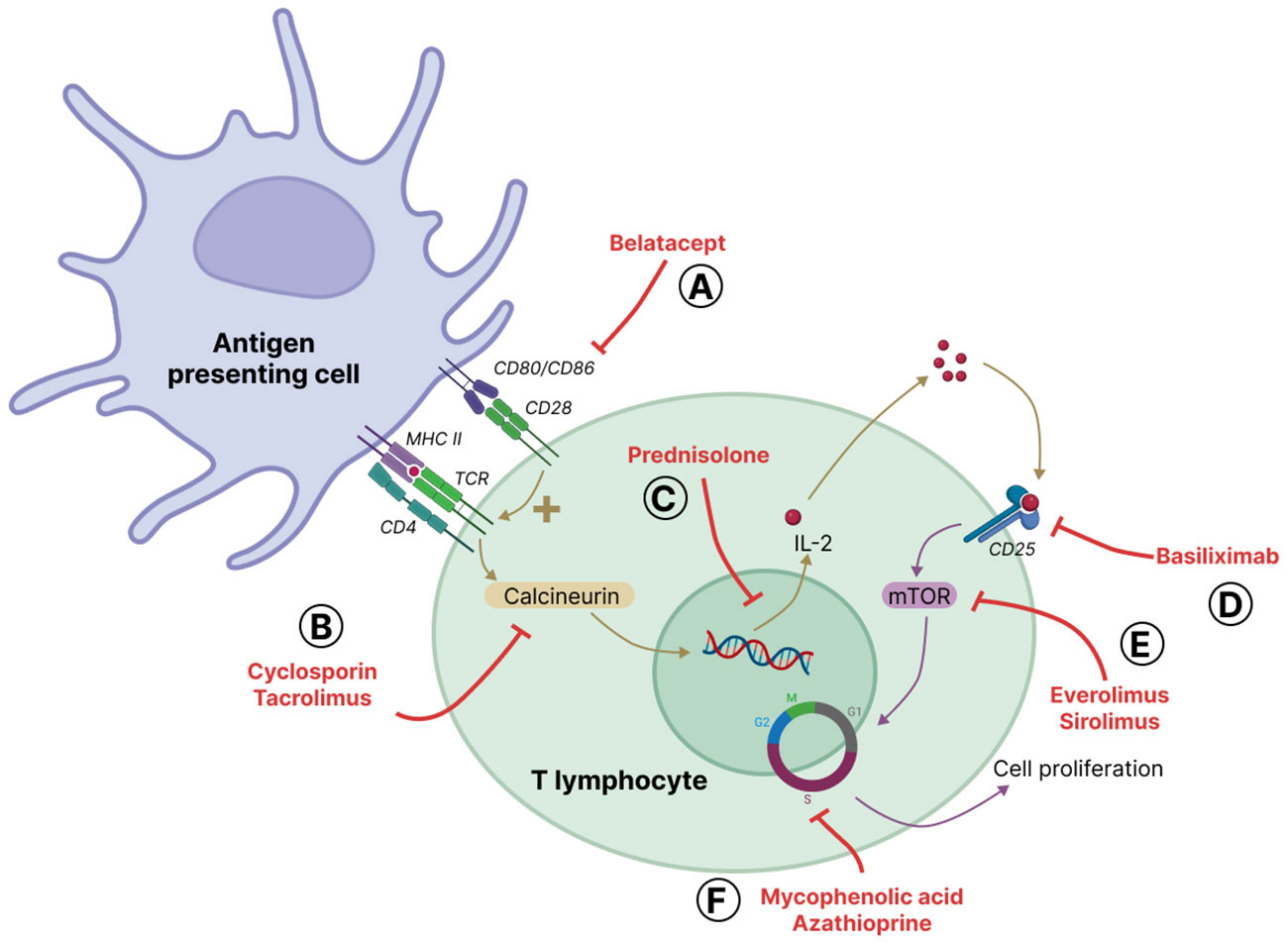
Main immunosuppressive drugs and their sites of action. Alloreactive T cells are activated by antigen presenting cells (APC) through two complementary signals: activation of T cell receptor (TCR) through antigen presentation bound to major histocompatibility complex (MHC) and co-activation *via* the interaction of CD28 on T cells and CD80/CD86, also known as B7, on the surface of APC. TCR activation leads to calcium-dependent signaling, including the activation of the phosphatase calcineurin that dephosphorylates the transcription factor NFAT that in turn mediates the synthesis of several cytokines. A major cytokine produced is the autocrine interleukin-2 (IL-2) that binds to its receptor on the surface of T cells, the alpha-chain of which, CD25 is also regulated by NFAT. IL2-receptor activation leads to mammalian target of rapamycin (mTOR)-mediated cell cycle entry and T cell proliferation. The major classes of immunosuppressive drugs available are **(A)** belatacept that blocks CD28 costimulatory action, **(B)** calcineurin inhibitors, *i.e.* cyclosporin and tacrolimus, **(C)** corticosteroids (mainly prednisolone) that inhibit the transcription of genes, including the one coding for IL-2, as well as intra-cellular calcium mobilization **(D)** basiliximab that blocks IL2-receptor engagement by IL2, **(E)** mTOR inhibitors, *i.e.* sirolimus and everolimus, and **(F)** antiproliferative agents, i.e. azathioprine and mycophenolic acid, that prevent cell cycle progression by inhibiting nucleotide synthesis. Of note, belatacept and basiliximab are known as biological immunosuppressive agents; belatacept is a fusion protein mimicking CTLA4 and acting as a mock receptor for CD80/CD86; basiliximab is a monoclonal antibody directed against CD25.

Immunosuppressive drugs are used to mitigate the immune response in solid organ transplantation. The major classes of approved maintenance immunosuppressive drugs are calcineurin inhibitors (cyclosporine and tacrolimus), glucocorticoids (primarily prednisolone), the co-signal inhibitor belatacept, mammalian target of rapamycin (mTOR) inhibitors (sirolimus and everolimus) and antiproliferative agents (azathioprine and mycophenolic acid) (**Figure 4**) (Wiesner and Fung, 2011). All of them inhibit T cell proliferation and activation. Indeed, proliferation of T cells depend on a three-step cascade that precedes cell cycle entry. The first step starts with the respective interaction of T cell receptor (TCR) and B7 on the surface of T cell with MHC presenting antigen and CD80/CD86 expressed by APC; the induced signaling cascade in T cell involves the calcium-dependent phosphatase calcineurin. Belatacept, a fusion protein, mimics the inhibitory receptor CTLA4 for B7, and thus blocks the co-stimulation signal (Rangel, 2010). Calcineurin inhibitors block the calcineurin-dependent NFAT signaling pathway (Wang et al., 2022). The second step is the transcription of different cytokine genes including that of the autocrine factor IL-2; glucocorticoids also block this step. IL-2 is secreted and binds to its receptor CD25 expressed by activated T cells and thus provokes the third step, *i.e.*, a mTOR-dependent signaling cascade leading to cell cycle entry. Basiliximab, a monoclonal antibody against CD25 inhibits IL2 binding to its receptor, and the mTOR inhibitors blocks the third step of T cell activation (Zaza et al., 2014). Antiproliferative agents inhibit cell cycle by blocking a T and B cell specific pathway of nucleotide synthesis required for the S phase of cell cycle. Thus, antiproliferative agents are also inhibitors of B cell proliferation. In addition to their role on T cell activation and proliferation, glucocorticoids are known to affect B cell, neutrophil, macrophage, DC and NK cell activation and/or proliferation (Allison and Eugui, 2000; Muscari et al., 2022).

To prevent graft rejection and promote graft tolerance in the long term, immunosuppressive drugs are commonly used in different combinations of calcineurin inhibitors, anti-proliferative agents or mTOR inhibitors, with or without glucocorticoids (Wiesner and Fung, 2011; Hartono et al., 2013; Enderby and Keller, 2015; Claeys and Vermeire, 2019; Fuehner et al., 2019). The treatment of established graft cellular rejection usually consists in the use of a pulse therapy of corticosteroids and increased doses of calcineurin inhibitors, and/or of anti-thymocyte antibodies in case of severe or resistant rejection (Cooper, 2020). The most common treatment of ABMR consists in plasmapheresis to deplete circulating alloreactive antibodies, and either steroid pulse or, less frequently, rituximab to limit B-cell proliferation and activation (Alasfar et al., 2023; Rostaing et al., 2023).

### 3.2 Adverse effects

The use of powerful antirejection agents may lead to opportunistic infections, reactivation of latent organisms, and post-surgery complications. The pattern of common infections after solid organ transplantation varies according to the immunosuppressive therapy and environmental exposure (Patel and Paya, 1997; Singh and Limaye, 2015; Fishman, 2017). These infections include bacterial (*e.g.*, *Clostridium* spp., *Enterobacteriaceae*, *Streptococcus pneumoniae*), viral (*e.g.*, cytomegalovirus, Epstein-Barr virus, respiratory viruses), and fungal (*e.g.*, *Aspergillus species*, *Candida species*, *Pneumocystis* spp.) species (Karuthu and Blumberg, 2012; van Delden et al., 2020). Whenever necessary, transplant patients receive antimicrobial drugs in addition to their immunosuppressive regimen.

The immunosuppressive drugs may also induce malignancies by reducing the vigilance of immune cells towards potentially carcinogenic cells. This have been pointed out in primary or secondary immunodeficiency disorders (Ilham et al., 2023). A population-based study of transplant recipients observed a 2-fold overall increased risk of cancer, ranging from non-Hodgkin lymphoma to transplant-related cancer (kidney, liver, and lung) (Engels et al., 2011). In addition, a recent study showed that kidney transplant recipients with ultra-long-term survival of more than 20 years have an increased risk of developing post-transplant lymphoproliferative disorder and renal cell carcinoma (Fuhrmann et al., 2022).

Other adverse effects (see European Medicines Agency Summary of Product Characteristics) are also observed with immunosuppressive drugs. Indeed, cyclosporine and tacrolimus are nephrotoxic (Griffin and Nelson, 2016) and favor post-transplant diabetes mellitus, hypertension as well as a large spectrum of cardiovascular diseases including heart failure, and coronary artery disease. Corticosteroids, calcineurin and mTOR inhibitors also favor post-transplant diabetes mellitus. Mycophenolic acid entails gastro-intestinal disorders such as nausea, vomiting and diarrhea. It also induces cell apoptosis and architectural remodelling of the lower gastro-intestinal tract, exhibiting colitis-like or inflammatory bowel disease-like patterns (Selbst et al., 2009).

In addition, immunosuppressive drugs as well as antimicrobial agents, frequently given to transplant patients, induce gut microbiota modifications due to their antimicrobial and immunosuppressive properties. The comparison of gut microbiome from kidney transplant patients and healthy controls revealed that age, body mass index (BMI), estimated glomerular filtration rate (eGFR) and medications (mycophenolate mofetil, antibiotics and proton-pump inhibitors) were the main co-variables explaining variations in the gut microbiota of kidney transplant patients (Swarte et al., 2020). However, it is quite difficult to identify a typical alteration induced by each type of immunosuppressive drug in transplant patients, as they are treated with combined therapy. However, efforts have been made to characterize the contribution of each type of drug, mainly using preclinical models (Gabarre et al., 2022). Indeed, administration of glucocorticoids, tacrolimus or mycophenolic acid to preclinical models result in alteration of the composition of gut microbiota and the fecal concentration of bacterial metabolites (Wu et al., 2018; Zhang et al., 2018; Taylor et al., 2019; Jardou et al., 2021). The most relevant clinical contribution has been reported in the paper by Swarte and colleagues, which supports that mycophenolic acid is one of the main drivers of changes occurring in the gut microbiome of kidney transplant recipients. In this work, they found that the gut microbiome of kidney transplant patients more than one-year post-transplantation, is significantly different from that of healthy population, containing more Pseudomonadota (ex Proteobacteria), less Actinomycetota (ex Actinobacteria), with a functional loss of butyrate-producing bacteria. The use of mycophenolic acid and antibiotics was associated with variation in the gut microbiome of kidney transplant patients and correlated with lower diversity (Swarte et al., 2020). Liver transplant recipients experiencing TCMR presented a lower diversity index, which represents microbial diversity, in the post-transplant as compared to the pre-transplantation period (Kato et al., 2017). At the phylum level, an increase in *Pseudomonadota* and *Actinomycetota* and a decrease in *Bacillota (ex Firmicutes)* were observed (Kato et al., 2017). Moreover, the paper by Swarte and colleagues provide some evidence that age and BMI are variables that are associated with alteration in the microbiota of kidney transplant patients (Swarte et al., 2020).

## 4 The gut microbiota

### 4.1 Composition of the gut microbiota

The gut microbiota is composed of a diverse and complex microbial community, which contributes to human health. This community of microorganisms includes bacteria, archaea, viruses and fungi that are distributed throughout the gastro-intestinal tract (Kho and Lal, 2018). Among the bacterial species, four main phyla represent 98% of the gut microbiota in healthy adults, of which *Bacillota* (60-80%) and *Bacteroidota* (15-25%) are the dominant bacterial phyla, followed by *Pseudomonadota* and *Actinomycetota* (Jandhyala et al., 2015; Zhang et al., 2019). The diversity of the gut microbiota depends on host age, genetic parameters, physiological status and health condition, and on exposure to various environmental factors including diet and medication (Falony et al., 2016; Zhernakova et al., 2016; The Milieu Intérieur Consortium et al., 2019). The gut microbiota exerts important effects on host homeostasis, including digestion of dietary fibers, synthesis of essential vitamins, maintenance of the intestinal barrier integrity as well as education and homeostasis of the immune system (Macfarlane and Macfarlane, 2012; Yu et al., 2012; Sommer and Bäckhed, 2013; Takiishi et al., 2017; Okumura and Takeda, 2018). The gut microbiota is also involved in drug metabolism, potentially leading to the activation, inactivation or toxicity of medicines (Li et al., 2016; Guo et al., 2019; Zimmermann et al., 2019).

Divergence from the ‘normal’ composition and function of the gut microbiota, called dysbiosis, can occur in pathological conditions such as chronic gastro-intestinal diseases, cardiovascular diseases, metabolic disorders, neurological disorders, carcinogenesis and others (Guinane and Cotter, 2013; Petersen and Round, 2014; Lloyd-Price et al., 2016; Schroeder and Bäckhed, 2016). Changes in the composition of microbiota and alterations of the production of microbial metabolites are referred to as structural and functional dysbiosis, respectively. Increasing data shows that dysbiosis is not only associated with disease occurrence but also partly responsible for disease development.

### 4.2 Pathways for digestive SCFA production

The gut microbiota produces a large amount of metabolites that influence host homeostasis (Fan and Pedersen, 2021; Wang et al., 2023). There are short-chain fatty acids (SCFA), represented by acetate, propionate, and butyrate, produced from undigested dietary fiber. Bacterial members of the gut microbiota generate carbon and energy from fermentable carbohydrates that resist digestion by host metabolic enzymes. This process is termed “prebiosis” (Hugenholtz et al., 2013). These fermentable carbohydrates are metabolized in the colon by the microbiota via the glycolytic and pentose phosphate pathways that generate deoxy-hexoses and hexoses from starch, cellulose and fructans, and pentoses from xylans and pectins, respectively (Cummings, 1981; Macfarlane and Macfarlane, 2003; Deleu et al., 2021). The main SCFA produced by the gut microbiota are acetate (C2), propionate (C3) and butyrate (C4), in approximate proportions of 60:20:20 (Chambers et al., 2018). Formate (C1), valerate (C5), caproate (C6) and branched-chain fatty acids (*e.g.*, isobutyrate, 2-methyl-butyrate, and isovalerate) can also be produced by the gut microbiota (Macfarlane and Macfarlane, 2003; Heaney, 2020).

The most abundant phyla in the gastro-intestinal tract, *i.e.*, *Bacteroidota* mainly produce acetate and propionate, whereas *Bacillota* mostly release acetate, propionate and butyrate (Macfarlane and Macfarlane, 2003; He et al., 2020). Pathways for the production of acetate are commonly spread among bacteria species and this SFCA achieves the highest concentrations in the intestine (Louis and Flint, 2017), whereas more specific pathways and substrates have been described for propionate and butyrate production. *Bacteroidota* and *Negativicutes* (from *Bacillota* phylum) use the succinate pathway for propionate biosynthesis while *Lachnospiraceae* (from *Bacillota* phylum) use the propanediol pathway (Reichardt et al., 2014). Moreover, there are two pathways for butyrate synthesis mediated by specific enzymes: butyrate kinase for *Coprococcus eutactus* and *Coprococcus comes* species; or butyryl CoA and acetate CoA transferase for *Faecalibacterium prausnitzii*, *Eubacterium rectale* and *Roseburia intestinalis* species (Reichardt et al., 2014; Deleu et al., 2021).

### 4.3 SCFA transporters and receptors

**Figure 5:**
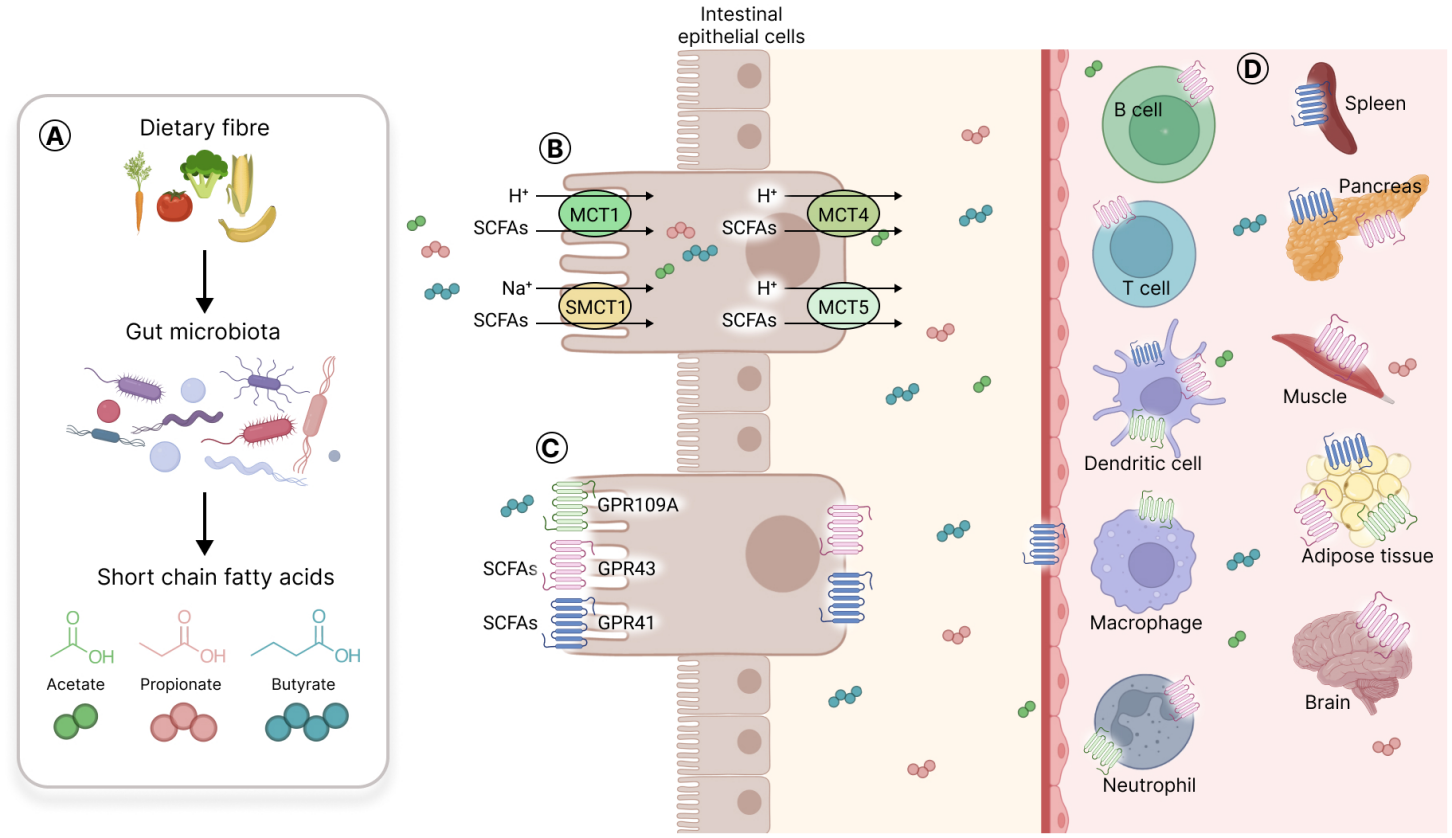
Short chain fatty acids production, transporters, and receptors. **(A)** Gut microbiota produces short chain fatty acids (SCFA) from fermentable carbohydrates that resist digestion by host metabolic enzymes. The three main SCFA produced are acetate, propionate, and butyrate. **(B)** SCFA can passively penetrate the mucosa or can be actively transported through SMCT1 or MCT1 located on the cell apical side, and MCT4 or MCT5 on the basal side of intestinal epithelial cells. **(C)** SCFA selectively activate three different G protein coupled receptors (GPR) on intestinal cells, namely GPR41, GPR43 and GPR109A; the last one being activated only by butyrate. **(D)** The SCFA receptors are expressed on gut epithelial cells, on immune cells (T cells, B cells, dendritic cells, macrophages, neutrophils) and on various other cell types in solid organs such as the spleen, the pancreas, the muscle, the adipose tissue and the brain.

SCFA can passively penetrate and pass through the apical membrane of digestive epithelial cells (Rechkemmer and von Engelhardt, 1988). SCFA can also be actively transported by sodium-coupled monocarboxylate transporter 1 (SMCT1) or monocarboxylate transporter 1 (MCT1) located on the apical side of digestive epithelial cells (Cuff et al., 2002; Miyauchi et al., 2004; Gill et al., 2005; Gupta et al., 2006). SCFA are used by colonocytes as a source of energy that provides 6 to 10% of their daily caloric requirements (Bergman, 1990). The remainder is transported through the basolateral membrane into the bloodstream through MCT4 and MCT5, located there (Gill et al., 2005; Bik et al., 2018) (**Figure 5**).

SCFA are selective agonists of three different G-protein coupled receptors (GPCR) in intestinal cells, namely, GPR41 (free fatty acid receptor 3 or FFAR3), GPR43 (free fatty acid receptor 2 or FFAR2) and, only for butyrate, GPR109A (hydroxycarboxylic acid receptor 2 or HCAR2) (Brown et al., 2003; Thangaraju et al., 2009; Zhao et al., 2018). These receptors are also expressed on various other cell types such as immune cells (*e.g.*, neutrophils, B and T cells, macrophages and DC) and endothelial cells, and in different organs and tissues such as the brain, adipose tissue, spleen and muscles (Le Poul et al., 2003; Vinolo et al., 2011; Li et al., 2017; van der Hee and Wells, 2021) (**Figure 5**). Of note, SCFA receptors have not been detected so far on NK cells.

In addition, some SCFA, particularly butyrate, have histone deacetylase (HDAC) inhibitory activity leading to epigenetic modification of the genes, allowing their differential expression (Licciardi et al., 2011). A well-known example of this pathway is the acetylation of the Foxp3 locus by butyrate in T cells leading to Foxp3 transcription factor expression and differentiation of regulatory T lymphocyte (Treg) (Arpaia et al., 2013).

## 5 SCFA-induced immunomodulation

SCFA produced by the gut microbiota are involved in the local and systemic regulation of immune functions. Overall, they have anti-inflammatory functions and they promote the differentiation and the function of immune cells with immunosuppressive properties (Zhang et al., 2019; Wang et al., 2023). Immunomodulation results either from their direct effect on immune cells or their indirect effect on non-immune cells, *e.g.*, gut epithelial cells or endothelial cells, that participate in immune reactions by facilitating the recruitment and the activation of immune cells (Li et al., 2018; Deleu et al., 2023).

### 5.1 Neutrophils

Neutrophils are the first immune cells to be attracted to an inflammatory site. They can be activated by pathogens to produce cytokines. These cytokines initiate the recruitment and activation of other immune cells, coordinating the overall immune response (Mayadas et al., 2014). Neutrophil express receptors for immunoglobulins and are activated by antibodies bound to antigens, leading to the production of cytokines and ROS and the secretion of granules. ROS production is known to be toxic to tissues while granule secretion may cause tissue damage or help tissue repair (Kruger et al., 2015; Wang, 2018). In the context of solid organ transplantation, neutrophils are typically the first type of leukocytes to infiltrate transplanted organs and to orchestrate local inflammation and possibly provoke tissue damage (Schofield et al., 2013; Scozzi et al., 2017).

*In vitro*, propionate and butyrate significantly reduce the production of pro-inflammatory molecules, such as TNF-α and cytokine-induced neutrophil chemoattractant-2 (CINC-2αβ) induced by the bacterial product lipopolysaccharide (Vinolo et al., 2011). Butyrate blocks microbial product-induced ROS production, whereas acetate favors the basal production of ROS (Vinolo et al., 2009). In addition, propionate and butyrate provoke caspase-8 and 9-dependent apoptosis of non-activated neutrophils and facilitate activated neutrophil apoptosis by inhibiting HDAC activity (Aoyama et al., 2010). Moreover, in a mouse model, acetate was protective against ischemia- and reperfusion-induced injuries, which was associated with a decreased number of activated neutrophils found in kidney tissue and a reduced level of oxidative stress in kidney cells (Andrade-Oliveira et al., 2015).

### 5.2 B lymphocytes

B lymphocytes play a key role in the humoral immune response and are involved in organ rejection by producing DSA. Moreover, B cells can affect the transplanted organs by interacting with and regulating T cells (Schmitz et al., 2020). Propionate and butyrate treatment inhibit immunoglobulin class-switch recombination and somatic hypermutation in B cells and diminish plasma cell differentiation (Sanchez et al., 2020). Moreover, acetate promotes B10 lymphocytes, a subpopulation of regulatory B cells that produce the potent Th1 inhibitory and anti-inflammatory cytokine IL10 (Kalampokis et al., 2013; Matsumoto et al., 2014; Daïen et al., 2021). In a mouse model, butyrate has been shown to induce B10 cell differentiation (Kim et al., 2021).

### 5.3 T lymphocytes

T lymphocytes are central in the immune response against foreign antigens occurring after solid organ transplantation. T lymphocytes recognize antigens presented by APC and either differentiate into cytotoxic cells, the Tc, or activate effector inflammatory cells that damage foreign tissue (Issa et al., 2010). SCFA modulates T lymphocyte differentiation and function through the inhibition of HDAC activity or *via* GPR binding (Wang et al., 2023). The most potent effect of SCFA on T cells is the promotion of Treg, a subpopulation with potent inhibitory function on Th cells and considered as the major actor of immune tolerance against self-antigens (Bayati et al., 2021). Of note, the potential use of Treg as an alternative or as a supplement to immunosuppressive therapies in organ transplantation to reduce graft rejection and avoid associated adverse effects has been discussed and investigated (Mathew et al., 2018; Sánchez-Fueyo et al., 2020; Juneja et al., 2022). *In vitro*, butyrate and to a lesser extent the other SCFA isovalerate and propionate, stimulate, *via* inhibition of HDAC, the generation of Treg (Arpaia et al., 2013; Furusawa et al., 2013; Kespohl et al., 2017; Verma et al., 2018). The positive regulation of Treg by butyrate was confirmed in a mouse model (Arpaia et al., 2013). In addition, in a mouse model of kidney transplantation, acetate and butyrate supplementation prolonged allograft survival by promoting tolerance towards graft (Wu et al., 2020). By depleting Treg or by using GPR43 deficient mice, the authors showed that the benefit of acetate supplementation resulted from the promotion of Treg differentiation through a GPR43-dependent pathway (Wu et al., 2020).

### 5.4 APC

APC, including DC and macrophages play a significant role in the immune response after transplantation. Macrophages may differentiate into pro-inflammatory cells with high microbicidal activity, called M1, or into anti-inflammatory cells with low microbicidal potential, known as M2 (Yunna et al., 2020). M1 macrophages are involved in acute and chronic inflammation, as well as in graft rejection. In contrast, M2 macrophages have a beneficial role in promoting Treg differentiation and immune tolerance towards the graft (Schmidt et al., 2016). Some authors propose to target DC and to modulate macrophages as a new treatment strategy to improve transplant outcomes (Merad et al., 2007; Panzer, 2022). Butyrate and propionate strongly inhibit human monocyte-derived DC activation by bacterial products (Nastasi et al., 2015). Butyrate has the same effect on murine bone marrow-derived DC: murine DC preincubated with butyrate favors the production of the anti-inflammatory cytokine IL10 by mouse splenocytes (Berndt et al., 2012). More recently, it was shown *in vitro* and *in vivo* that butyrate, through its HDAC inhibitory activity, imprints a potent anti-microbial activity during macrophage differentiation (Schulthess et al., 2019). Wang et al. (2020b) showed *in vitro* that propionate and butyrate suppress the pro-inflammatory M1 phenotype of macrophages while promoting the anti-inflammatory M2 phenotype (Wang et al., 2020b). They also demonstrated in a mouse model that the fermentable fiber inulin-induced reduction of alcoholic liver disease is associated with an increased intestinal content of propionate and butyrate and, in parallel, with the suppression of hepatic M1 macrophages and an increased number of M2 macrophages (Wang et al., 2020b). Another paper showed in a mouse model of kidney injury that acetate decreases the numbers of kidney infiltrating macrophages and kidney cell oxidative stress that may result from ROS production by macrophages (Andrade-Oliveira et al., 2015). In addition, *in vitro* butyrate reduces the production of inflammatory mediators (TNF-α, IL-6, inducible nitric oxide synthase) by macrophages (Ohira et al., 2013). Using high fiber diet in a mouse model of airways viral infection, Trompette et al. (2018) demonstrated that butyrate diminishes the ability of macrophages to produce the proinflammatory mediator C-X-C motif ligand 1 (CXCL1) and the subsequent airways neutrophil influx (Trompette et al., 2018). Finally, Singh et al. (2014), combining *in vitro* and *in vivo* experiments, brought evidence that butyrate activates GPR109A and promotes the production of IL10 by colonic macrophages, boosting Treg differentiation and reducing colon carcinogenesis (Singh et al., 2014).

## 6 Conclusion

In solid organ transplantation, graft rejection may occur within hours to years after surgery. This can happen through multiple mechanisms (Moreau et al., 2013). To manage the risk of graft rejection, patients are treated life-long with immunosuppressive drugs that dampen their immune system. However, immunosuppressive drug use is associated with serious adverse effects including severe intestinal lesions, metabolic diseases, cardiovascular disorders and cancer that significantly impair patient quality of life and affect graft outcome. After transplantation, most patients present with dysbiosis and some immunosuppressive drugs alter the production of SCFA (Swarte et al., 2020; Jardou et al., 2021). SCFA are essential metabolites for host homeostasis; through specific receptor activation and/or HDAC inhibition, SCFA regulate the local and systemic immune systems, directly by acting on neutrophil, B and T cells, macrophages, and DC and indirectly by affecting the ability of epithelial and endothelial cells to recruit and activate immune cells. Overall, in these cells, the microbial metabolites SCFA favor anti-inflammatory or immunosuppressive properties (Zhang et al., 2019). The decreased production of SCFA due to maintenance immunosuppressive drugs may thus lead to permanent low-level systemic inflammation with deleterious effect on the graft in the long run.

There is some experimental evidence that restoring a “normal” level of SCFA in pre-clinical models might be beneficial (Wu et al., 2020); exogenous SCFA could oppose systemic inflammation and reinforce the immunosuppressive effects of drugs, allowing lower doses hence mitigating their adverse effects.

Other potentially active gut microbial metabolites may be involved in the development of comorbidities and graft rejection, but require further investigation. For example, primary bile acids, which are converted to secondary bile acids by the gut microbiota, are important regulators of glucose and lipid homeostasis. Bile acids may also signal through specific receptors to regulate the immune system (Fiorucci et al., 2018). The gut microbiota is also involved in the production of branched-chain amino acids (*i.e.*, valine, isoleucine, and leucine), the alteration of which could lead to disturbances in protein synthesis, glucose and lipid metabolism, insulin resistance and immune disorders (Holeček, 2018). Tryptophan derivatives produced by some bacterial strains are involved in maintaining gut barrier homeostasis and improving glucose metabolism (Li et al., 2021) and could be of interest as and adjunctive therapy as they have complementary anti-inflammatory and immunosuppressive properties, including the ability to inhibit NK cell activation (Wang et al., 2023). Gut microbiota can also produce trimethylamines, which are converted to trimethylamine-N-oxide (TMAO), high levels of which are associated with cardiovascular disorders (Fadhlaoui et al., 2020).

Overall, observational and, further, interventional clinical studies are needed to better evaluate the benefit of SCFA and other gut microbiota-derived metabolites in transplantation and their synergistic effects with immunosuppressive drugs.

## Author contributions

MJ: Writing – original draft, Conceptualization, Writing – review & editing. CB: Writing – review & editing, Conceptualization. PM: Writing – review & editing, Conceptualization. NP: Writing – review & editing, Conceptualization. AD: Writing – review & editing, Conceptualization, Writing – original draft. RL: Writing – review & editing, Conceptualization, Writing – original draft.
